# Alteration of the bZIP60/IRE1 Pathway Affects Plant Response to ER Stress in *Arabidopsis thaliana*

**DOI:** 10.1371/journal.pone.0039023

**Published:** 2012-06-12

**Authors:** Sabrina Humbert, Sihui Zhong, Yan Deng, Stephen H. Howell, Steven J. Rothstein

**Affiliations:** 1 Department of Molecular and Cellular Biology, University of Guelph, Guelph, Ontario, Canada; 2 Plant Sciences Institute, Iowa State University, Ames, Iowa, United States of America; Instituto de Biología Molecular y Celular de Plantas, Spain

## Abstract

The Unfolded Protein Response (UPR) is elicited under cellular and environmental stress conditions that disrupt protein folding in the endoplasmic reticulum (ER). Through the transcriptional induction of genes encoding ER resident chaperones and proteins involved in folding, the pathway contributes to alleviating ER stress by increasing the folding capacity in the ER. Similarly to other eukaryotic systems, one arm of the UPR in Arabidopsis is set off by a non-conventional splicing event mediated by ribonuclease kinase IRE1b. The enzyme specifically targets mature bZIP60 RNA for cleavage, which results in a novel splice variant encoding a nuclear localized transcription factor. Although it is clear that this molecular switch widely affects the transcriptome, its exact role in overall plant response to stress has not been established and mutant approaches have not provided much insight. In this study, we took a transgenic approach to manipulate the pathway in positive and negative fashions. Our data show that the ER-resident chaperone BiP accumulates differentially depending on the level of activation of the pathway. In addition, phenotypes of the transgenic lines suggest that BiP accumulation is positively correlated with plant tolerance to chronic ER stress.

## Introduction

Eukaryotic cells have evolved elaborate mechanisms to maintain cellular homeostasis under changing environmental conditions. In particular, the ER stress response is elicited under conditions that compromise adequate protein folding in the ER. Studied in much detail in mammals, yeast and more recently in plants, the response contributes to alleviating ER stress through the activation of membrane-anchored stress transducers. Although the complexity varies depending on the organism, there are three main routes by which cells reduce the load of misfolded ER proteins resulting from ER stress: the first promotes the degradation of misfolded proteins through the 26S proteasome system in a process called ERAD (ER-associated degradation); the second route consists of slowing the rate of new protein synthesis and is mediated by PERK in mammalian cells; the third route involves increasing the protein folding capacity in the ER through the transcriptional activation of ER-resident chaperones and proteins involved in protein folding. All are part of the Unfolded Protein Response (UPR), a highly conserved response which, in plants, serves to mitigate damage caused by adverse environmental conditions. UPR has been described extensively in yeast and mammalian cells, where a non-conventional splicing event mediated by ribonuclease kinase Inositol Requiring Enzyme 1 (IRE1) has been shown to play a major role in ER stress perception and signalling (reviewed in [1]). Recently, a similar mechanism of IRE1-mediated RNA splicing was identified in Arabidopsis, establishing the existence of this mode of regulation in a plant system [2].

Arabidopsis has two IRE1 orthologs, IRE1a and IRE1b, which are structurally similar to their animal and yeast counterparts, despite a relatively low overall sequence homology. Protein localization experiments as well as *in vitro* evidence reported by two independent groups strongly suggested that plant IRE1s perform functions similar to their yeast and mammalian orthologs, the splicing of a mRNA encoding a stress induced transcription factor [3], [4]. This hypothesis was confirmed with the identification of bZIP60 mRNA as a target of IRE1-mediated splicing *in vivo* [2].

*Arabidopsis* bZIP60 was first identified by [5] who showed that the gene is induced by ER stress agents such as dithiothreitol (DTT). In unstressed seedlings, bZIP60 protein appears in full-length form localized to the ER. Following treatment with stress agents a smaller form of the protein was detected in nuclei [6]. The smaller form of the protein activates the expression of ER stress response genes such as ER-resident chaperone BiP (Binding Protein) [7]. Recently, bZIP60 mRNA was found to undergo splicing in response to treatment with DTT, in a manner similar to mammalian Xbp1 and yeast Hac1 [2]. The transcriptionally active form of bZIP60 protein results from the translation of a novel mRNA splice variant, lacking a 23-base intron. The splicing was attributed to IRE1, and while both IRE1a and b have ribonuclease activity in vitro, IRE1b is primarily responsible for the splicing reaction in seedlings. A brief survey of naturally occurring abiotic stress conditions revealed that heat treatment rapidly induces bZIP60 splicing, suggesting that the IRE1b/bZIP60 pathway is important in mediating plant response to abiotic stress such as heat.

This finding raised the question of the impact of the IRE1b/bZIP60 pathway on plant fitness under ER stress conditions. While in other systems, mutations in genes involved in the IRE1 branch of the UPR have been linked to various defects (for example [8], [9]), surprisingly no obvious phenotype has been reported in *Arabidopsis* mutants of the IRE1/bZIP60 pathway. In this study, we demonstrate that the pathway impacts various aspects of *Arabidopsis* growth and development under stress conditions.

## Results

### Alteration of the bZIP60/IRE1 pathway by mutant and transgenic approaches

**Figure 1:**
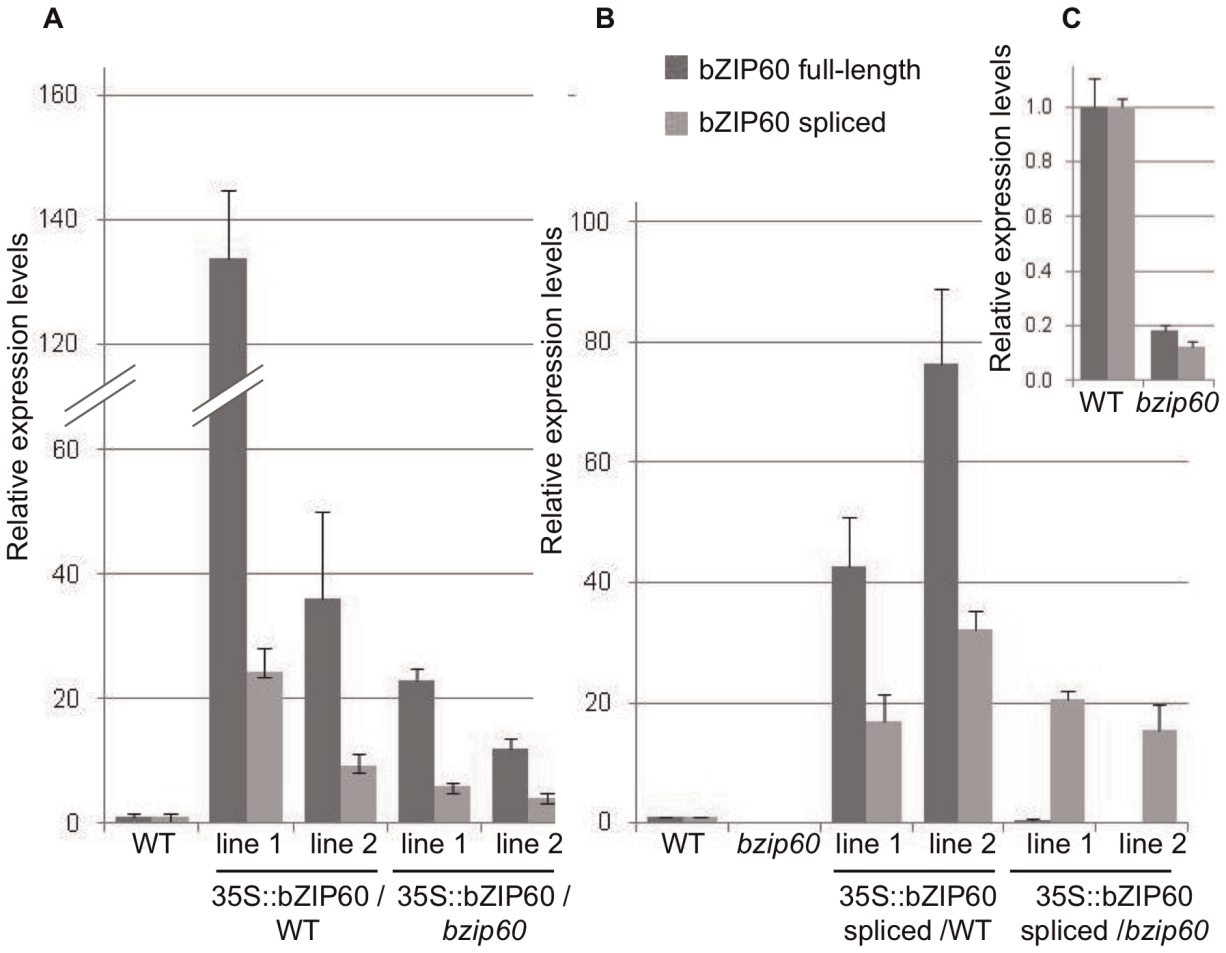
Expression of bZIP60 transcript variants in transgenic and mutant lines. Quantification by qRT-PCR of bZIP60 transcript variants relative to the Ubiquitin 5 reference gene in 8 d-old seedlings in lines over-expressing the full-length transcript (A) and in lines over-expressing the spliced version (B) in WT and *bzip60* mutant backgrounds. Graph C is a close-up view of the graph in B showing expression levels in *bzip60* mutant compared to WT. Two independent lines are shown for each transformed construct. Error bars = SE.

A *bzip60* mutant was first described by Iwata *et al.* [10]. The T-DNA insertion (salk_50204) disrupts the first exon of the gene and abolishes its transcription in homozygous plants. Under normal growth conditions, the mutant plants do not display any obvious defects compared to WT. Stable transgenic lines over-expressing bZIP60 full-length and spliced variants were generated in both WT and *bzip60* mutant backgrounds. Although transformation was carried out in parallel for both constructs in all backgrounds, obtaining transformants bearing the spliced construct proved to be challenging. More than 20 independent lines were recovered in each background for the full-length constructs while only two lines were obtained for the spliced constructs. Quantification of RNA transcripts derived from the transgenes by real-time PCR confirmed successful over-expression in all backgrounds (Figure 1). Specificity of each primer pair toward the full-length or spliced transcripts was verified by running dissociation curves in all experiments and analyzing PCR amplification products on agarose gels. It is interesting that over-expression of the full-length transcript resulted in increased amounts of spliced variant as well, even in the *bzip60* mutant background, suggesting that some proportion of the transgene undergoes splicing *in vivo* under nonstressed conditions (Figure 1-A). In addition, the data obtained for the lines over-expressing the spliced variant showed that the resulting protein is sufficient to induce the transcription of its own transcript, as illustrated by the drastic over-expression of the full-length variant in the WT background (Figure 1-B). This was not the case in the bzip60 mutant background, where no full-length variant was induced, due to the presence of the T-DNA insertion at the bZIP60 locus.

**Figure 2:**
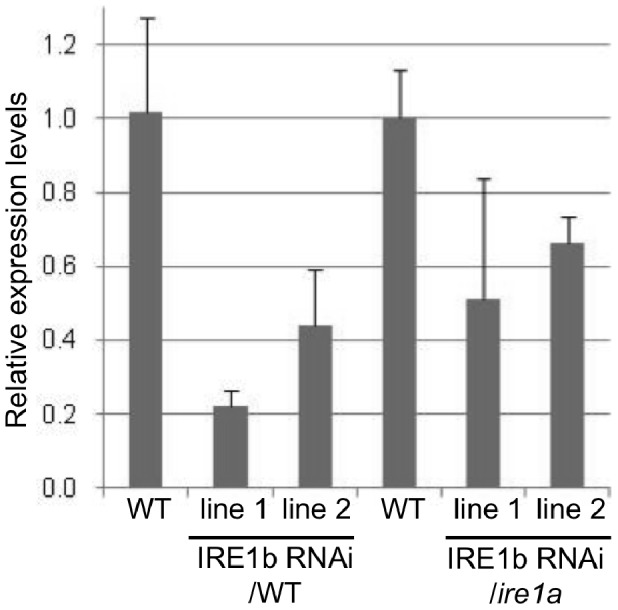
Expression of IRE1b transcript in RNAi lines. Quantification by qRT-PCR of IRE1b transcripts relative to the Ubiquitin 5 reference gene in 8 d-old seedlings in lines expressing the IRE1b RNAi construct in WT and *ire1a* mutant backgrounds. Two WT controls are displayed on the graph to account for the data obtained in separate assays. Two independent lines are shown for each transformed construct. Error bars = SE.

**Figure 3:**
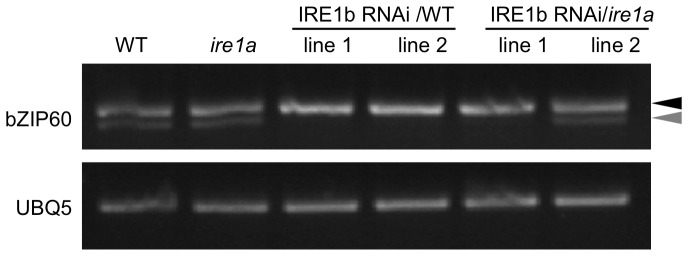
bZIP60 splicing in RNAi lines after heat stress treatment. Semi-quantitative RT-PCR showing bZIP60 splice variants in WT, *ire1a* mutant and IRE1b RNAi lines in WT and *ire1a* mutant backgrounds. Two independent lines are shown for each transformed construct. The full length and spliced variant are respectively indicated by black and grey arrowheads.

An *Arabidopsis ire1a* null mutant was characterized previously by Lu and Christopher [11]. The T-DNA insertion (salk_018112) is located in the third exon and successfully disrupts the expression of full-length IRE1a transcript. However, ER-responsive genes are normally induced in this mutant [11] and bZIP60 splicing still occurs under ER stress conditions [2], suggesting that IRE1a is either not functional or redundant with its IRE1b homolog. An *ire1b* mutant (salk_018150) with a T-DNA insertion in the exon was found to be homozygous lethal [11]. Recently another mutant (sail_238_F07) was found with a T-DNA insertion in an intron which abolished full-length IRE1b expression [2], however, that line produces a partial transcript which may have phenotypic consequences [12]. Therefore, we took an RNA interference approach to generate plants with diminished IRE1b expression. Constructs targeting IRE1b for degradation were transformed in both WT and *ire1a* mutant backgrounds as described in Materials and Methods. Successful knock-down of IRE1b transcript levels was demonstrated by quantitative real-time PCR. Significantly lower expression of IRE1b was obtained using this strategy in both backgrounds (Figure 2). In addition, we verified that bZIP60 splicing was impaired in those lines under heat stress treatment (Figure 3). bZIP60 splice variant could not be detected in 3 out of the 4 lines selected for this analysis, confirming that IRE1b is necessary for the splicing event to occur as reported previously [2]. The line that did not show a significant inhibition of the splicing activity also displayed higher levels of IRE1b transcript, which could account for this the result.

### BiP protein accumulation correlates with the activation/inhibition of the IRE1/bZIP60 pathway

Although *bzip60* does not show any obvious developmental defects when grown under standard conditions, Iwata et al. [10] found pronounced transcriptional differences in the mutant compared to WT when treated with the ER stress inducer tunicamycin. Among the 129 genes that were induced more than threefold by the treatment in WT, 54 were induced at significantly lower levels in the *bzip60* mutant, suggesting that the absence of the gene might produce visible effects under stress conditions. In particular, induction of BiP3 transcript was much reduced in the mutant compared to the WT under stress conditions, and this observation held true at the protein level as well [10].

BiP is the most abundant chaperone in the ER and was identified as one of the major target genes elevated by UPR [13], [14]. BiP belongs to the HSP70 (Heat Shock Protein 70) family, a group of heat-induced proteins highly conserved in the plant kingdom [15]. BiP proteins are involved in various processes such as the regulation of endosperm proliferation in *Arabidopsis* [16] or the accumulation of seed storage proteins in rice [17]. Besides heat, BiP is upregulated in response to salt stress [18] and over-expression of BiP confers drought tolerance in soybean and tobacco [19], [20]. In *Arabidopsis*, BiP is encoded by three genes, named BiP1 to BiP3. While BiP1 and 2 transcript levels are constitutively quite high, BiP3 is usually expressed at lower levels but is induced to a much larger extent under stress conditions. Notably, BiP3 has been shown to be one of the main transcriptional targets of active bZIP60 transcription factor [7], [21]. Nevertheless, the transcript is still induced in the *bzip60* mutant and seems to be dependent on other ER stress transducers such as bZIP28 [22]. Making a direct link between the IRE1/bZIP60 pathway and BiP3 transcript levels is therefore not straightforward. By contrast, monitoring the resulting BiP protein levels might provide a better view of the folding capacity in the ER and more accurately capture the stress status of the plant.

**Figure 4:**
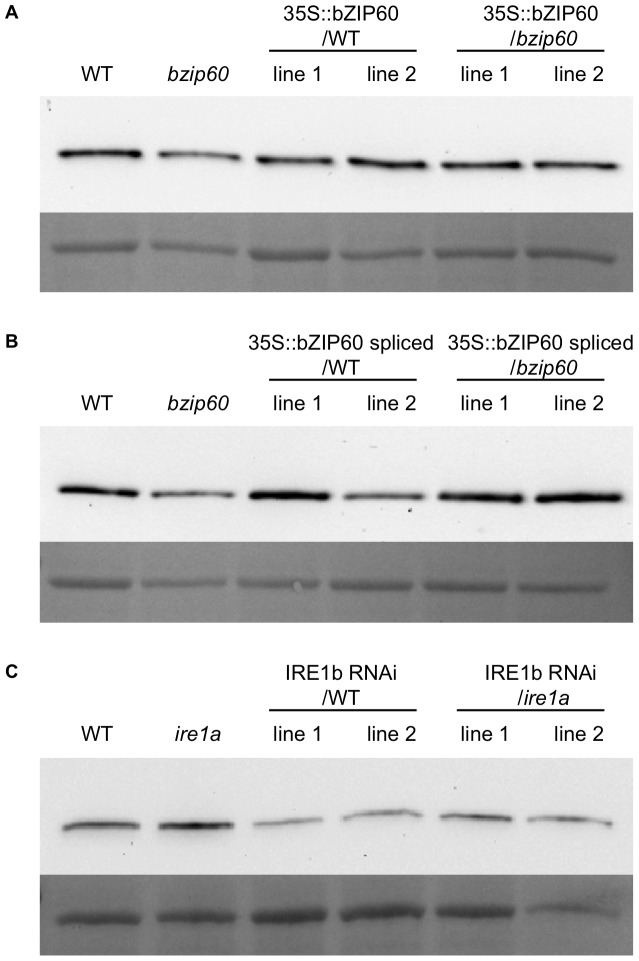
BiP protein accumulation in transgenic and mutant lines altering the bZIP60/IRE1 pathway. Western blots revealing BiP levels in total protein extracts from 8 d-old seedlings treated with 2 mM DTT for 8 h. The membrane was stained with Ponceau red and shows the large subunit of Rubisco as a loading control. A. WT, bzip60 mutant and transgenic lines over-expressing bZIP60 full-length in WT and *bzip60* backgrounds. B. WT, *bzip60* mutant and transgenic lines over-expressing bZIP60 spliced in WT and bzip60 backgrounds. C. WT, *ire1a* mutant and IRE1b RNAi lines in WT and *ire1a* backgrounds. Two independent lines are shown for each transformed construct.

In this study, we used a commercially available BiP monoclonal antibody that had been successfully used in previous publications to monitor BiP levels in plants and more specifically in *Arabidopsis* [23]–[26]. The single band observed on Western blots around 70–75 kDa corresponds to any of the three BiP protein isoforms present in Arabidopsis (predicted molecular weights of 73.6, 73.5 and 75.1 kDa). As previously reported [10], BiP protein was found to be highly induced in response to DTT treatment in WT by contrast with the *bzip60* mutant where induction could barely be detected (Figure S1). We then assessed BiP levels in the transgenic lines over-expressing the bZIP60 variants. As shown in Figure 4, BiP protein levels in transgenic plants were at least equal to WT after 8 h of DTT treatment. This was the case for lines over-expressing both the full-length (Figure 4A) or the spliced (Figure 4B) bZIP60 transgene. Similarly, we tested transgenic RNAi lines with diminished IRE1b levels. RNAi seedlings treated with DTT for 8 h displayed significantly lower BiP accumulation in both WT and *ire1a* mutant backgrounds while the *ire1a* mutant did not prevent BiP induction (Figure 4C).

### Up- or down-regulation of the IRE1/bZIP60 pathway affects plant tolerance to ER stress

As was observed with the *bzip60* mutant, none of the bZIP60 transgenic lines displayed obvious phenotypes when grown on soil under standard conditions. Similarly, the IRE1b RNAi lines did not show any visible defects under unstressed conditions. However, it seemed reasonable to test whether phenotypes of the mutant or transgenic lines might be altered under chronic ER stress conditions. Root growth in WT plants was significantly reduced when seedlings were grown on agar plates with DTT concentrations as low as 0.5 mM (Figure S2-A). Germination of WT seeds was also inhibited by DTT, although the effect only became significant at slightly higher concentrations (>5 mM) (Figure S2-B). At 10 mM DTT, germination was completely inhibited. DTT is easily oxidized in contact with air, which results in a gradual depletion of its reducing power. We therefore completed the assay within a few days after sowing as it was clear that seedlings started recovering from the treatment after one week, suggesting that the effects of the reducing agent had significantly weakened by that time.

**Figure 5:**
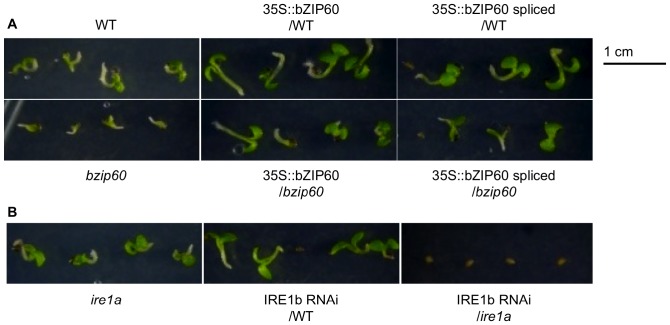
Phenotype of transgenic and mutant seedlings grown for 7 days on MS medium supplemented with 2.5 mM DTT. A. WT, *bzip60* mutant and transgenic lines over-expressing full-length or spliced bZIP60 variants in WT and *bzip60* backgrounds. B. *ire1a* mutant and IRE1b RNAi lines in WT and *ire1a* backgrounds. One representative line is shown for each transformed construct.

**Figure 6:**
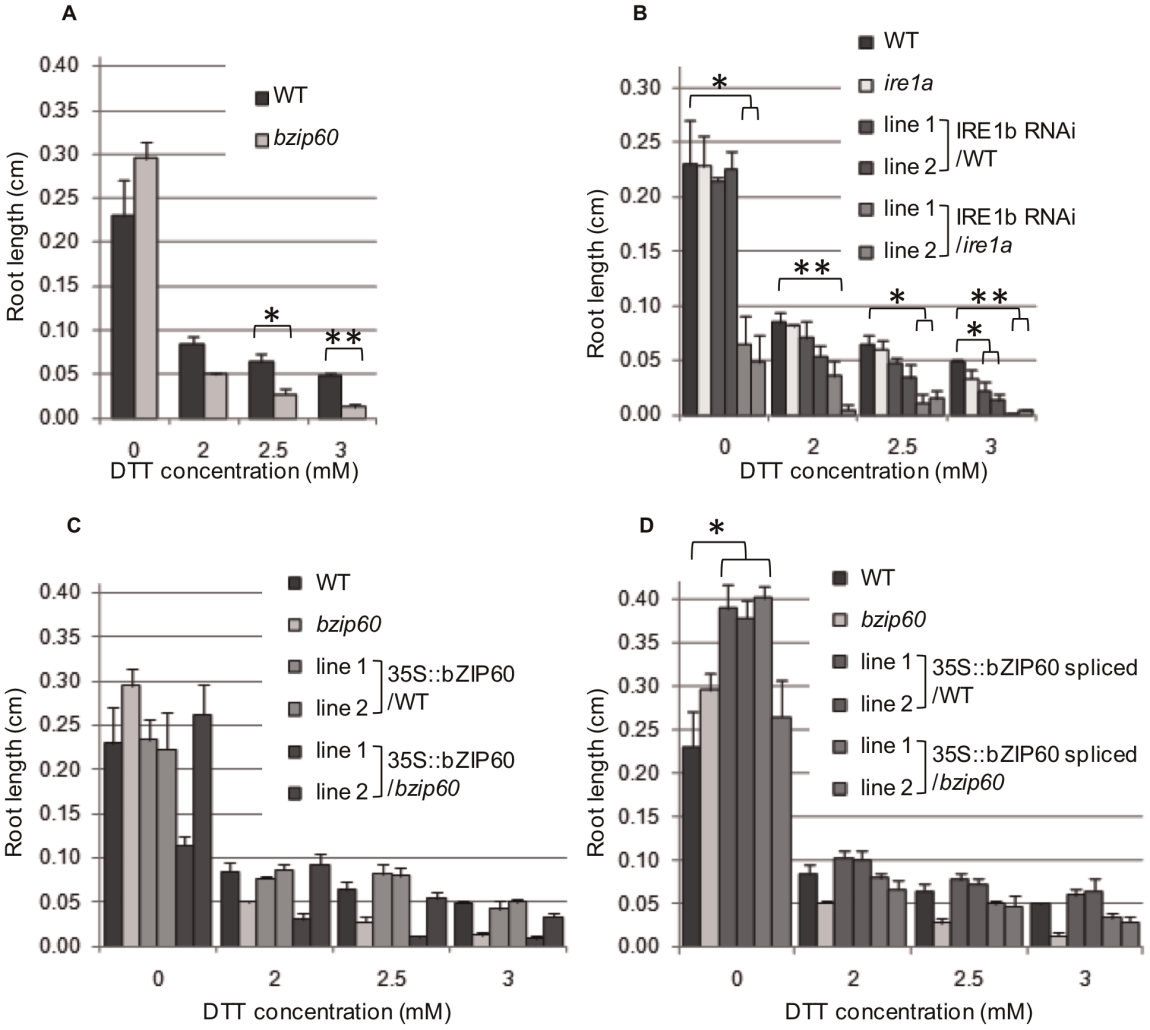
Root growth in response to DTT treatment in mutants and transgenic lines. Root length measured three d after sowing on MS medium supplemented with indicated concentrations of DTT in WT, mutant (A), IRE1b RNAi lines (B), lines over-expressing bZIP60 full-length (C), and lines over-expressing bZIP60 spliced (D). Values correspond to averages of biological replicates and corresponding standard errors. Student's t-tests were performed to assess the significance of the results for the different lines compared to WT. Statistically significant comparisons are indicated on the graphs with * and ** denoting respectively a p-value ≤0.05 and 0.01.

As expected, *bzip60* displayed a similar root growth phenotype compared to WT when grown on standard MS medium, *i.e.* unstressed conditions (Figure S3 and Figure 6A). However, the mutant was more sensitive to DTT treatment than WT. At 2.5 mM DTT, root growth was inhibited in both WT and *bzip60* seedlings, but the mutant was affected to a larger extent and was barely able to germinate (Figure 5A). The difference in root length between WT and *bzip60* seedlings was statistically significant at 2.5 mM DTT and above (Figure 6A). By contrast, bZIP60 over-expression lines were found to perform at least as well as the WT under DTT stress, and better than the *bzip60* mutant. While root lengths were not statistically different compared to WT (Figure 6C and D), seedling growth was visibly enhanced (Figure 5A). IRE1b RNAi lines were also assessed for their response to DTT treatment. When grown on soil under standard unstressed conditions, the plants were slightly smaller than WT but the difference was not statistically significant. On standard MS plates however, the IRE1b RNAi transgenic lines in an *ire1a* background had shorter roots than either the WT or *ire1a* mutant (Figure 6B). The presence of DTT in the medium resulted in even more pronounced differences between WT and these transgenic lines, which could barely germinate on 2.5 mM DTT (Figure 5B). In general, RNAi lines generated in both backgrounds were found to be more severely affected by the treatment as illustrated by the root length measurements, and the differences with WT were statistically significant at 3 mM DTT for all lines (Figure 6B). Altogether, these results demonstrate that alterations of the IRE1/bZIP60 pathway produce effects at the plant level under stress conditions. The effects are largely correlated with the up or down regulation of the pathway.

## Discussion

The present study focused on the consequences of manipulating the IRE1/bZIP60 pathway in *Arabidopsis*. Our data suggest that upregulation of bZIP60 enhances seedling tolerance to chronic ER stress, while down-regulation of IRE1a and b leads to negative effects. Levels of chaperone BiP were observed to follow a comparable trend, with a significant decrease in BiP induction when IRE1b was down-regulated. The correlation between BiP levels and plant tolerance to stress suggests that the protein is important in protecting plants against the deleterious effects of stress treatment. This supports previous findings suggesting a role for BiP in enhancing plant tolerance to abiotic stresses such as drought [19], [20]. It also suggests that plant tolerance to stress possibly results from the protective effects of UPR induction.

The expression of BiP3 is largely dependent on its transcription by active bZIP60 [7] and active bZIP60 is able to activate its own expression through an ERSE (ER response element)-like present in its promoter [5]. The present study confirmed that expression of the spliced version of the transcript auto-regulates its own expression *in vivo*. BZIP60 splicing thus constitutes a powerful molecular switch turned on under stress conditions. By contrast, the role of the full-length protein variant, which appears to be constitutively produced in the cell, remains elusive and our data did not allow drawing any conclusion in that regard. Although our transgenic approach attempted to discriminate between the two variants by over-expressing either the full-length or the spliced transgene in the *bzip60* mutant background, it resulted in the appearance of the spliced variant in both cases even under non-stressed conditions, complicating further investigation of the specific function of full-length bZIP60. A role for unspliced Xbp1as a negative regulator of spliced Xbp1 has been found in mammalian cells [27] and it will be interesting to determine whether its plant counterpart performs a similar function.

Chen and Brandizzi [12] have examined the effects of *ire1a* and *ire1b* mutants on germination and root growth in *Arabidopsis* and have obtained similar results to those reported here. However, they reported that the *ire1b-2* mutant used in their study is not a null mutation and that partial transcripts are found. Since the *ire1b* null mutant is thought to be an embryonic or reproductive lethal, the survivability of *ire1b-2* implies that partial transcripts might still be functional. Although the IRE1b RNAi transgenics that we have generated here likely lead to the destruction of whole transcripts, the RNAi approach only knocks down, but does not entirely knock out transcript accumulation. This has been used to an advantage in our study by providing us with lines with various levels of IRE1b gene expression. Chen and Brandizzi [12] did not look at the effect of overexpression of the full-length and spliced form of bZIP60 on *Arabidopsis*. By doing so, we found not only that overexpression of spliced bZIP60 autoregulates its own gene, but also that IRE1b RNAi in an *ire1a* background completely abolishes root growth under stress condition, while the *bzip60* mutant still displays some reduced growth. This suggests additional roles for IRE1A and B that do not involve bZIP60.

The study of ER stress response in plants was first described in the corn mutant *floury-2*, which shows surprisingly elevated levels of BiP and other ER-resident chaperones due to the aberrant accumulation of á-zein in the ER [28]. Since then, it has become clear that the UPR is involved in a variety of processes. In particular, a link between UPR and plant response to environmental stress is emerging [18]-[20], [29]. While protein folding is clearly affected by stresses such as heat, the process is also easily disturbed under conditions inducing a high demand for protein synthesis. This is the case for example during dark to light transitions, and it has been shown that exposure to light induces the UPR most likely to accommodate the increase in protein synthesis [30]. Another example of this phenomenon is during viral infection, where the UPR was recently found to be induced [31] and will likely be found to be important for other stress response pathways.

## Materials and Methods

### Genetic material and growth conditions

*Arabidopsis thaliana* (ecotype Columbia) seeds were stratified at 4°C for 3 d before germination. Unless indicated otherwise, plants were grown under continuous white light (∼100 ìmol m-2.s-1) at 23–25°C in LA4 soil or on Murashige and Skoog (MS) medium (1× MS salts, 1% sucrose, 0.8% agar) supplemented with the indicated amount of DTT. Mutants for *bzip60* (SALK_050203) and *ire1a* (SALK_018112) were obtained from the Arabidopsis Biological Resource Center. Transgenic lines were generated by Agrobacterium-mediated transformation as previously described [32]. T0 seeds were selected on MS medium supplemented with the appropriate antibiotic. T2 seedlings were selected again on antibiotic medium and the segregation ratio for most lines was >3∶1 (antibiotic resistant: sensitive). T3 plants were grown on soil, genotyped individually and checked for segregation on antibiotic-supplemented medium. Non-segregating T2 seed stocks (T3 plants) were used in all assays.

### Constructs and sequences

Full-length bZIP60 sequence was PCR-amplified using primers bZIP60F (5′-ATGGTACCATGGCGGAGGAATTTGGAAGCATA-3′) and bZIP60R (5′-TAGGATCCTCATCACGCCGCAAGGGTTAAG-3′), and cDNA synthesized from WT seedling RNA. Spliced bZIP60 sequence was PCR-amplified using primers bZIP60F and bZIP60splR (5′-TAGGATCCCTACTACTCCCGAGCCCGTTTAG-3′), and cDNA synthesized from DTT-treated seedling RNA. Both sequences were inserted into the pCHF1 binary vector using KpnI and BamHI sites. The vector allowed selection of transformants using gentamycin and expression of the transgenes in plant under the control of the 35S promoter. Both constructs were transformed in WT and *bzip60* mutant backgrounds.

The RNAi (RNA interference) strategy was pursued using the pHANNIBAL/pART27 system described by Gleave [33] and Wesley et al. [34]. An RNAi expression cassette was first created in intermediate vector pHANNIBAL. The cassette contained the gene-specific RNAi target region cloned in sense and anti-sense directions and separated by an intron, under the control of the 35S promoter. The expression cassette was then transferred to pART27 binary vector allowing kanamycin selection of plant transformants. Sequences targeting regions 187-385 and 1636-1885 of the IRE1b transcript were selected for their specificity and cloned using primers IRE1b-187F/R (5′-TGCTCGAGAGAGGATCTGCACTACTTG-3′/5′-AGTCTAGAGAGAGGATCTGCACTACTTG-3′) and IRE1b-385F/R (5′-GCAAGCTTCTGTGAAGACATATCCAC-5′-GCGGTACCTTCTGTGAAGACATATCC-3′) or IRE1b-1636F/R (5′-TTCTCGAGCGGTTAAGCGTCTAGTACAATC-3′/5′-ATTCTAGAGCGGTTAAGCGTCTAGTACAATC-3′) and IRE1b-1885F/R (5′-GCAAGCTTCTCAAAGATTGGGTTTATCTG-3′/5′-GCGGTACCTCAAAGATTGGGTTTATCTG-3′). The resulting constructs were transformed in both WT and *ire1a* mutant backgrounds.

### Real-time polymerase chain reaction

Total RNA was extracted using TRI-Reagent (Sigma-Aldrich) following the manufacturer's instructions. Samples were DNase treated using RQ1 RNase-free DNase (Promega) and quantified using a Nanodrop 2000c spectrophotometer (ThermoScientific). One µg was reverse transcribed using qScript cDNA SuperMix (QuantaBiosciences). The Real-Time PCR mix was prepared using PerfeCTA SYBR Green I mix (QuantaBiosciences) following the manufacturer's instructions. PCR amplification was performed on a 7300 RealTime PCR system (Applied Biosystems). Primer pair efficiency was determined by generating standard curves where cDNA dilutions ranged from 1∶1 to 1∶32. In all assays, 1∶4 dilutions of cDNA were used and each data point corresponded to the average of two technical replicates on the plate. Relative gene expression levels were assessed using Ubiquitin 5 (AT3G62250) as the reference gene and WT as the reference treatment. Calculations were performed taking into account reference gene primers and target primers efficiencies. Primers bZIP60F4 (5′- GAAGGAGACGATGATGCTGTGGCT-3′) and b60UB1 (5′-GCAGGGATTCCAACAAGAGCACAG-3′) were used to detect bZIP60 full-length variant. Primers bZIP60F4 and b60SB2 (5′-AGCAGGGAACCCAACAGCAGACT-3′) were used to detect bZIP60 spliced variant. Primers UBQ5F (5′-CTTGAAGACGGCCGTACCCTC-3′) and UBQ5R (5′-CGCTGAACCTTTCCAGATCCATCG-3′) were used to detect Ubiquitin 5. The values on the graphs correspond to 2 to 3 biological replicates per independent transgenic line and error bars show the standard error among those biological replicates.

### Protein extraction and Western blotting

Eight day-old seedlings grown vertically on MS medium were transferred into liquid MS medium supplemented with 2 mM DTT for 8 h. The tissue was frozen in liquid nitrogen and homogenized at 4°C in 1 ml extraction buffer per gram of fresh tissue. The extraction buffer was composed of 100 mM HEPES (pH 7), 20% glycerol, 1 mM EDTA (pH 8), 0.1% Triton-X 100 (final), 10 mM β-mercaptoethanol, and 0.1 mg/ml PMSF (phenylmethanesulphonyl fluoride). The mixture was immediately centrifuged at 12,200 g for 15 min at 4°C and the supernatant was transferred into a fresh tube for further analysis. Protein concentration was determined using a Bradford assay (Bio-Rad Protein Assay, 500-0006) and bovine serum albumin was used as a calibration standard. Protein extracts were resolved on a 10% SDS-PAGE gel (Protogel, National Diagnostics) using a Mini-Protean apparatus (Bio-Rad) and transferred to a nitrocellulose membrane (iBlot, Invitrogen). Ponceau staining was performed prior to antibody hybridization to verify equal loading and adequate transfer of proteins to the membrane (Ponceau S, #7170, Sigma-Aldrich). A primary antibody raised against recombinant spinach Hsc70 was used to monitor Arabidopsis BiP protein (SPA-818, Stressgen, Enzo Life Sciences) along with an HRP-linked anti-mouse antibody (ECL mouse IgG, NXA931NA934, GE Healthcare). Detection was performed using an enhanced chemiluminescence kit (ECL Plus, RPN2124, GE Healthcare).

### Arabidopsis root length measurements and germination

Seedlings germinated on MS medium supplemented with varying amounts of DTT were grown vertically for the indicated periods of time. Petri dishes were then scanned at 600 dpi resolution and root lengths were determined using ImageJ software (Rasband, W.S., ImageJ, U. S. National Institutes of Health, Bethesda, Maryland, USA, http://imagej.nih.gov/ij/, 1997–2011). Germination was defined as the emergence of the root. For each concentration of ER stress agent tested, four Petri dishes served as biological replicates that allowed calculating the average value of 5 to 10 seedlings each. The data shown on the graphs correspond to averages and standard errors for those biological replicates.

## Supporting Information

Figure S1 **BiP fails to be induced in DTT-treated bzip60 mutant seedlings.** Western blot showing BiP levels in total protein extracts from 8 d-old WT and *bzip60* seedlings treated with 2 mM DTT (+) or mock solution (−) for 4 and 8 h. The membrane was stained with Ponceau red and shows the large subunit of Rubisco as a loading control.
(TIF)

Figure S2 **Effect of DTT on root growth and germination.** Germination rates and root lengths were measured in WT seedlings 1, 3 and 6 d after sowing on DTT-supplemented MS medium. Error bars = SE.
(TIF)

Figure S3 **Phenotypes of transgenic and mutant lines.** A. WT, *bzip60* mutant and transgenic lines grown for 7 days on MS medium and over-expressing bZIP60 full-length or spliced variants in WT and *bzip60* backgrounds. B. *ire1a* mutant and IRE1b RNAi lines in WT and *ire1a* backgrounds. One representative line is shown for each transformed construct.
(TIF)
