# Supplementary figures and images for: Alteration of the bZIP60/IRE1 Pathway Affects Plant Response to ER Stress in Arabidopsis thaliana
Source: PLoS One. 2012 Jun 12;7(6):e39023. doi: 10.1371/journal.pone.0039023 (PMC3373542; doi:10.1371/journal.pone.0039023)

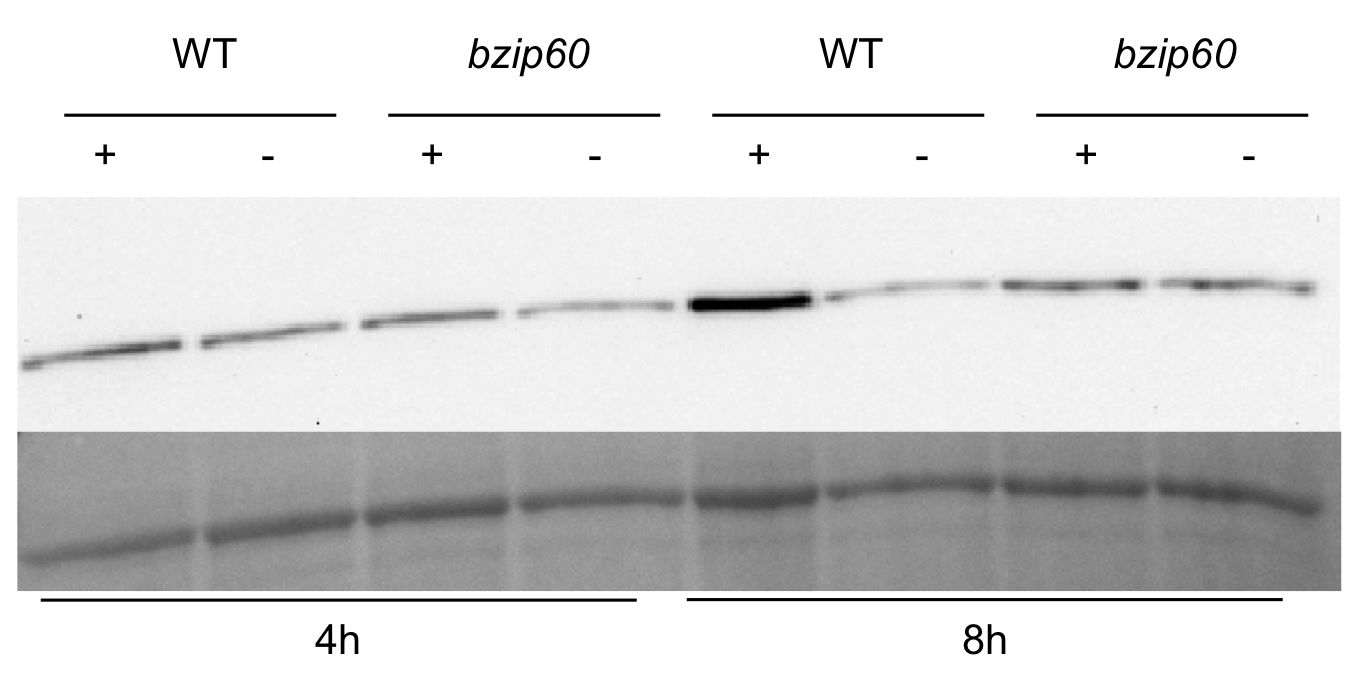

Supplement: Figure S1 — BiP fails to be induced in DTT-treated bzip60 mutant seedlings. Western blot showing BiP levels in total protein extracts from 8 d-old WT and bzip60 seedlings treated with 2 mM DTT (+) or mock solution (−) for 4 and 8 h. The membrane was stained with Ponceau red and shows the large subunit of Rubisco as a loading control. (TIF) [file pone.0039023.s001.tif]

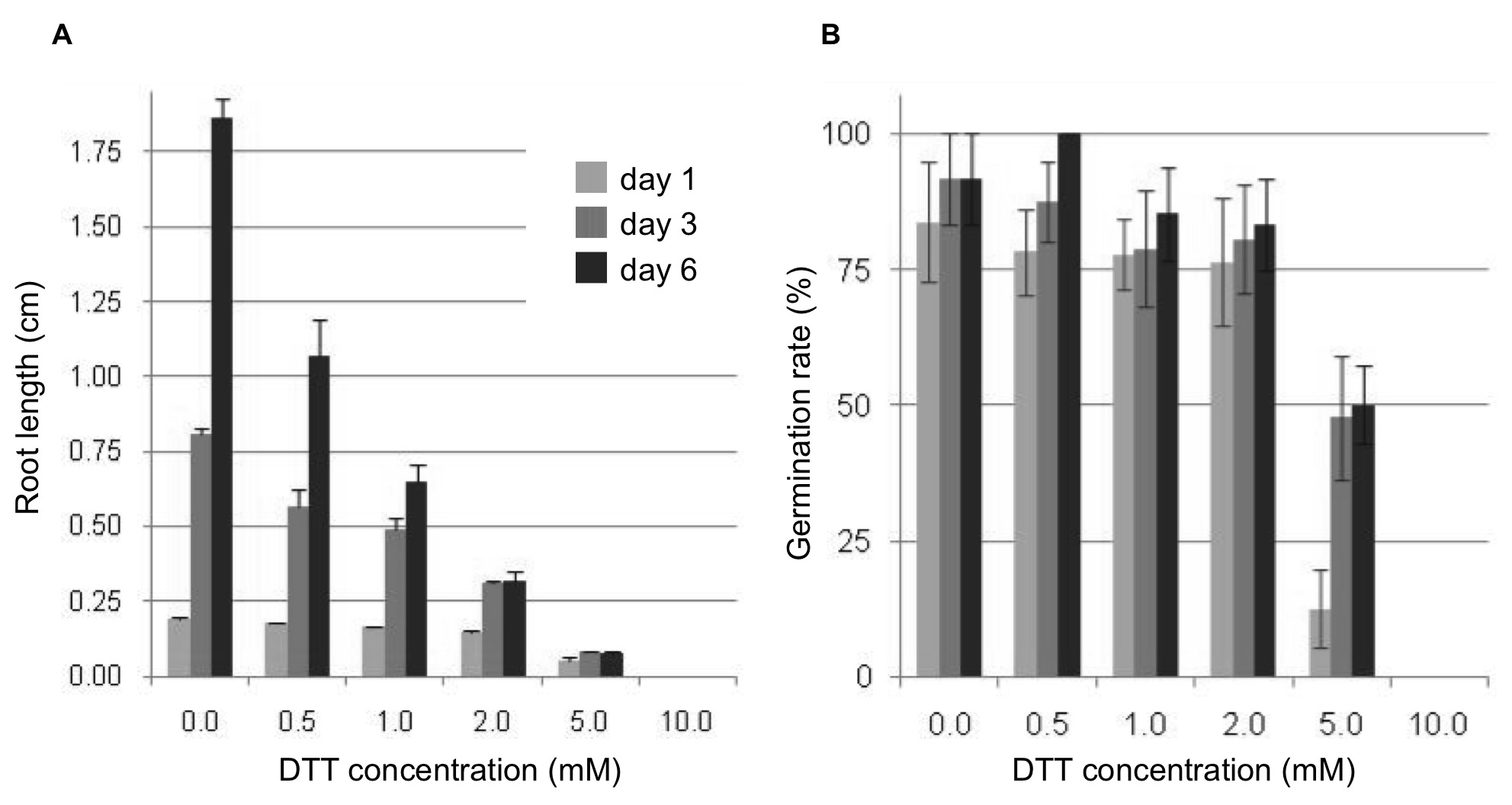

Supplement: Figure S2 — Effect of DTT on root growth and germination. Germination rates and root lengths were measured in WT seedlings 1, 3 and 6 d after sowing on DTT-supplemented MS medium. Error bars = SE. (TIF) [file pone.0039023.s002.tif]

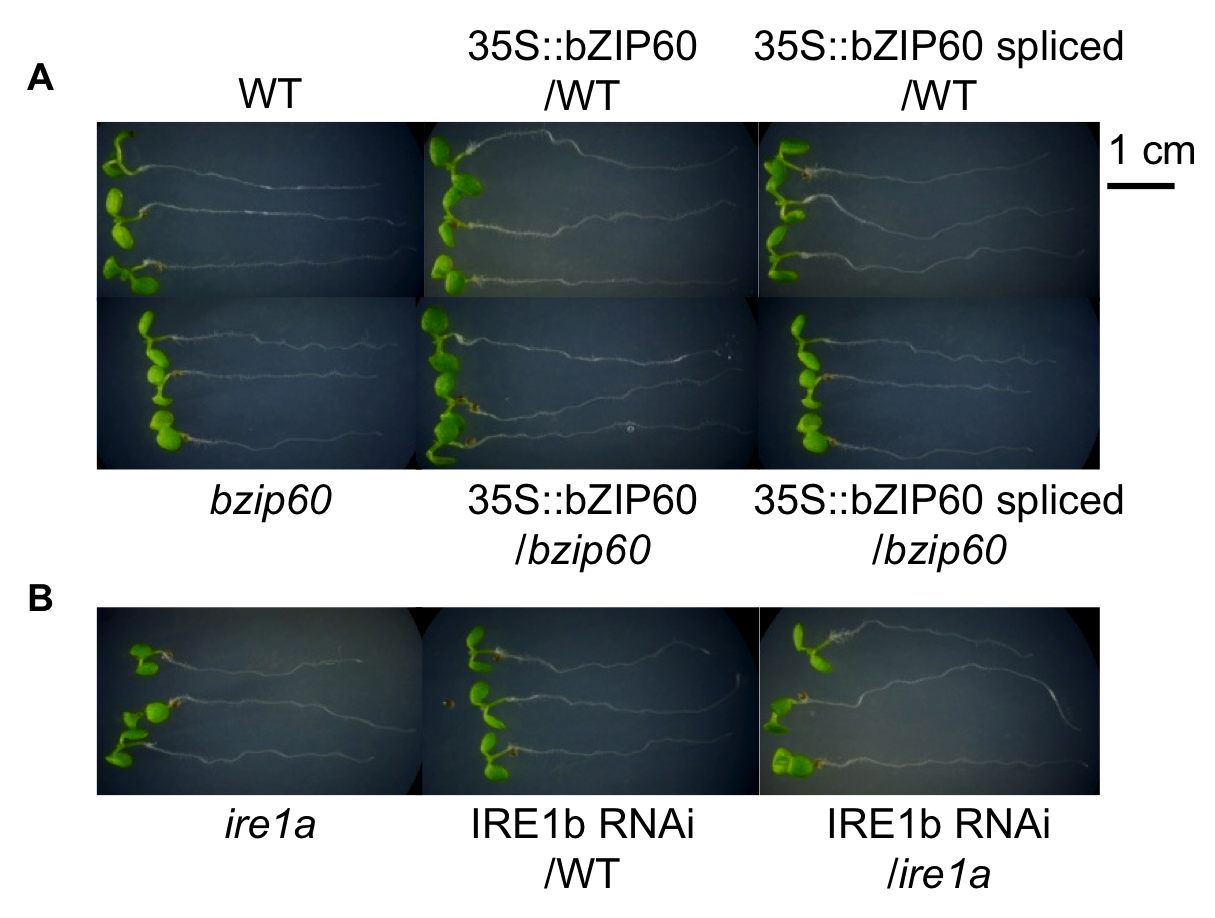

Supplement: Figure S3 — Phenotypes of transgenic and mutant lines. A. WT, bzip60 mutant and transgenic lines grown for 7 days on MS medium and over-expressing bZIP60 full-length or spliced variants in WT and bzip60 backgrounds. B. ire1a mutant and IRE1b RNAi lines in WT and ire1a backgrounds. One representative line is shown for each transformed construct. (TIF) [file pone.0039023.s003.tif]
